# Inhibiting KSHV replication by targeting the essential activities of KSHV processivity protein, PF-8

**DOI:** 10.1002/jmv.29958

**Published:** 2024-10

**Authors:** Jennifer Kneas Travis, Lindsey M. Costantini

**Affiliations:** 1 Department of Biological and Biomedical Sciences, North Carolina Central University, Durham, North Carolina, USA; 2 Integrated Biosciences (INBS) Doctoral Program, North Carolina Central University, Durham, North Carolina, USA

**Keywords:** antiviral targets, DNA processivity factors, KSHV, viral DNA replication

## Abstract

Kaposi’s Sarcoma Herpesvirus (KSHV) is the causative agent of several human diseases. There are no cures for KSHV infection. KSHV establishes biphasic lifelong infections. During the lytic phase, new genomes are replicated by seven viral DNA replication proteins. The processivity factor’s (PF-8) functions to tether DNA polymerase to DNA, so new viral genomes are efficiently synthesized. PF-8 self-associates, interacts with KSHV DNA replication proteins and the viral DNA. Inhibition of viral DNA replication would diminish the infection within a host and reduce transmission to new individuals. In this review we summarize PF-8 molecular and structural studies, detail the essential protein-protein and nucleic acid interactions needed for efficient lytic DNA replication, identify future areas for investigation and propose PF-8 as a promising antiviral target. Additionally, we discuss similarities that the processivity factor from Epstein-Barr virus shares with PF-8, which could promote a pan-herpesvirus antiviral therapeutic targeting strategy.

## 1 | INTRODUCTION

Gamma-herpesviruses are part of the Herpesviridae family and include the Murine Herpesvirus-68 (MHV-68), Herpesvirus Saimiri (HVS), Rhesus Macaque Rhadinovirus (RRV), Epstein-Barr virus (EBV), and Kaposi’s Sarcoma Herpesvirus (KSHV).^1,2^ All these viruses encode 118–170 kilobase double stranded DNA (dsDNA) genomes and establish lifelong infections.^2,3^ Despite the similarities between gamma-herpesviruses, they each have their own unique characteristics. For example, HVS does not cause disease in its natural host, but can in other new world primates such as owl monkeys and marmosets in a captivity setting.^2,4,5^ Herpesvirus diversity extend to variability in sequences, receptor preference, disease pathogenesis and cancer development.

KSHV is the most recently discovered human gamma-herpesvirus and was identified in 1994, now 30 years ago.^6^ KSHV is the causative agent of multicentric Castleman’s disease, primary effusion lymphoma, Kaposi’s sarcoma (KS), and KSHV inflammatory cytokine syndrome.^7–10^ After primary infection is established, KSHV like all herpesviruses can exist in a latent or lytic phase.^11^ The switch from latent to lytic depends on viral, host, and environmental factors.^12,13^ Once the virus undergoes lytic reactivation, the majority of viral genes are expressed, and the viral genome is replicated. The KSHV genome encodes its own set of lytic DNA replication proteins. The KSHV DNA replication proteins work in concert to form the replisome and efficiently replicate viral genomes. Targeting and disrupting genome replication, a key step in the lytic replication cycle, would prevent the new infectious virions from being produced and decrease the spread of KSHV. This strategy has been implemented by targeting and interfering with either the viral DNA polymerase or helicase primase in related human herpesviruses.^14,15^ For example, nucleoside analogs such as acyclovir target the viral DNA polymerase and are commonly used to treat Herpes Simplex Virus-1 and 2.^16^ Even though similar DNA polymerase inhibitors have been investigated as treatment options for KSHV, limited efficacy has been observed and there are still no effective treatments.^17^ One approach to discover new antiviral targets is to explore alternative strategies to inhibit lytic DNA replication, such as inhibitors targeting the accessory DNA replication proteins. Specifically, an antiviral therapy aimed at eliminating the functions of the processivity factor (PF) could be a promising strategy and has been shown effective for a subset of viruses.^18–21^

**FIGURE 1:**
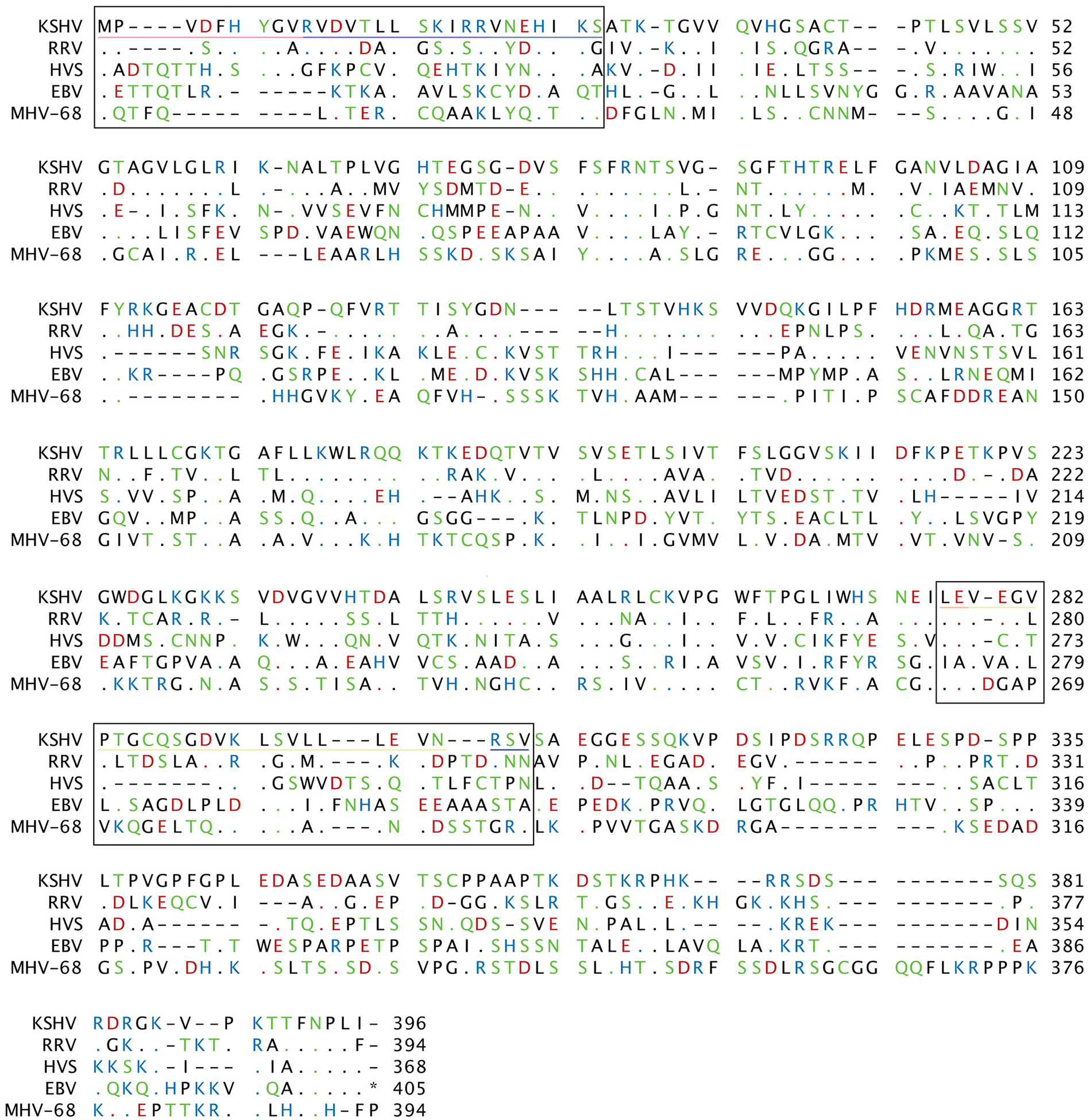
Sequence alignment of gamma-herpesviruses processivity factor protein sequences. Amino acid alignments for KSHV, RRV, HVS, EBV, and MHV-68 processivity factors. Amino acids are coloring corresponds with hydrophobic residues as black, hydrophilic residues as green, acidic residues as red and basic residues as blue. Dots indicate conserved residues. Boxes denote key KSHV PF-8 domains, residues 1–27 and 277–304. Line colors denote interaction domains: dimerization (fuchsia, medium slate blue, light salmon, and pale goldenrod), dsDNA (fuchsia, medium slate blue, and pale goldenrod), and DNA polymerase (medium slate blue, slate gray, light salmon, pale goldenrod). Alignment was created in CLC Workbench. dsDNA, kilobase double stranded DNA; EBV, Epstein-Barr virus; HVS, Herpesvirus Saimiri; KSHV, Kaposi’s Sarcoma Herpesvirus; MHV-68, Murine Herpesvirus-68; PF, processivity factor; RRV, Rhesus Macaque Rhadinovirus.

The primary function of PFs is to tether DNA polymerase to DNA so long strands of DNA can be synthesized.^22^ PFs have auxiliary functions including inhibiting host immune responses, interfering with DNA repair mechanisms, and activating transcription.^23^ The percent amino acid similarity of gamma-herpesvirus PFs compared to KSHV (GenBank: GQ994935.1) for RRV (GenBank: AAD21393.1), HVS (GenBank: CAA45682.1), EBV (GenBank: CAA24844.1), and MHV-68 (GenBank: AAB66449.1) are 52.2%, 30.1%, 25.4%, and 27.6%, respectively (Figure 1 and Table 1). While EBV is the only other identified human gamma-herpesvirus, it shares the least sequence similarity to KSHV. Due to the higher sequence similarities between MHV-68, HVS, and RRV, they are often used as a model system to study gamma-herpesviruses and KSHV.^3,4,24,25^

There are ongoing efforts towards engineering in vivo KSHV infection and oncogenic models that more closely resemble the characteristics of KSHV and recently a transgenic mice model was created to study KS that will allow for treatments and vaccines to be evaluated.^26^ Much of what is understood about KSHV PF (PF-8) is based on experiments using in vitro models, either with purified proteins or cell-based approaches. The objectives of this review are to (1) summarize the current knowledge of PF-8, specifically, the key protein domains and molecular interactions that underly PF-8 roles in lytic DNA replication, (2) offer new perspectives to support PF-8 as a viable antiviral target and (3) discuss outstanding questions for future studies to further define PF-8’s functional protein domains, DNA binding sites and protein-protein interactions required for efficient viral DNA replication.

## 2 | VIRAL DNA REPLICATION DURING LATENT AND LYTIC PHASES

The gene expression patterns and mechanisms of viral DNA replication differ between the latent and lytic phases.^11^ There are four main gene expression stages: latent, immediate early, early, and late.^27^ During latency, ORF73 (LANA-1), ORF72 (viral cyclin), and ORF71 (K13/vFlip) are primarily expressed.^28^ During latency the viral episome is maintained by mitosis via host cell machinery.^11,12^ Immediate early, early, and late genes are primarily expressed during the lytic phase.^29^ Immediate early genes typically encode for transcription factors and regulators such as ORF50.^30^ ORF50 encodes for the replication and transcription activator (RTA) protein and has been shown to be expressed within 10 h of reactivation.^11,13^ In addition, immediate early genes can trigger expression of early genes such as other DNA replication proteins.^28^ Early genes are necessary for viral genome replication and viral protein production. Late genes are expressed after replication and encode structural proteins.^30^ During the lytic phase the newly synthesized viral genome is packaged to form new virions.^11,13^

KSHV encodes three origins of replication, one latent and two lytic.^9^ The latent origin of replication has terminal repeats that allow for episome persistence.^31^ The two KSHV lytic origin sequences (oriLyt) are commonly known as left and right oriLyt and have a GC-rich repeat sequence, two long AT palindromes, an imperfect AT-rich palindrome, three TATA boxes, and binding motifs for AP-1, ATF, SP-1, and C/EBP.^9,32^ The left oriLyt is positioned between ORFK4.2 and ORFK5 while the right is between ORFK12 and ORF71.^31,33^ In addition to the lytic origins, there are seven essential viral proteins (gene) for lytic DNA replication: RTA (ORF50), single stranded DNA (ssDNA) binding protein (ORF6), DNA polymerase (ORF9), primase-associated factor (ORF40/41), helicase (ORF44), primase (ORF56), and PF-8 (ORF59).^32,34^ Although K8 (K-bZIP) is not considered a crucial DNA replication protein, it has a role in promoting lytic DNA replication.^30^

KSHV lytic DNA replication is believed to utilize a rolling circle mechanism where concatemeric DNA is produced and then cleaved.^31,35^ Other herpesviruses are thought to be replicated by the rolling circle mechanism, recombination, or a combination of the two.^36–38^ There are two suggested mechanisms of formation of the pre-initiation complex. The first mechanism proposes that RTA and K8/K-bZip bind to the oriLyt first and then recruit additional replication proteins. The second mechanism proposes that RTA, K8/K-bZip, and other replication proteins, including PF-8, associate and then bind to the oriLyt. In addition, the pre-initiation complex also includes of several cellular proteins such as topoisomerases.^35^ Wang et al. infers the proposed second mechanism is taking place since RTA and K8 coprecipitated with PF-8, ssDNA binding protein, and RTA in the absence and presence of ethidium bromide (EtBr) indicating that this complex is not mediated by DNA, since EtBr would likely diminish DNA-mediated protein binding.^31^

## 3 | KSHV DNA PF, PF-8

Most PF-8 studies utilized purified protein or cell lysates due to the limited availability of KSHV animal models. Purified proteins can be produced from in vitro transcription translation (IVT), bacterial, yeast, insect, or mammalian systems by various methods. An advantage of experiments using purified protein is the protein of interest can be studied alone or in combination with other purified components in a simplified system. Cell based approaches that isolate lysates from cell lines allow for proteins to be studied in a complex cellular environment. While infected cells provide the advantage of a biological relevant system to study the impacts of both cellular and viral components and the interplay of virus and host cell proteins.^39,40^ The collection of previous studies provide the basis for PF-8 candidacy as a potential drug target.

### 3.1 | Bacterial PF-8 structure and domains

**FIGURE 2:**
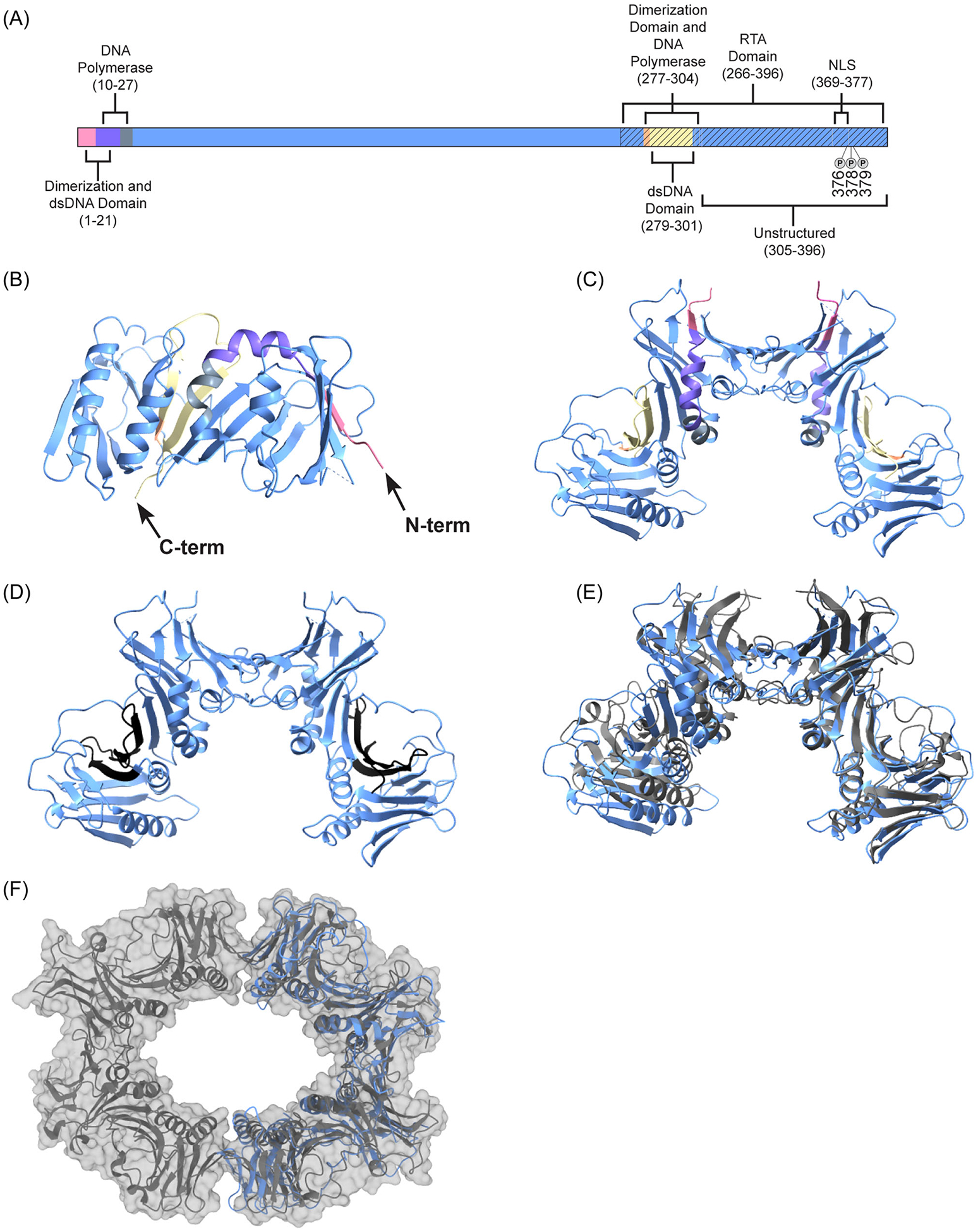
Domains, regions, and conformations of PF-8 and BMRF1. (A) The different domains and regions of PF-8 relative to the primary sequence that have been experimentally determined. (B, C) 3-Dimensional cartoon diagram of the monomer and dimer (cornflower blue) of PF-8 color coordinated with Panel A for the dimerization (fuchsia, medium slate blue, light salmon, and pale goldenrod), dsDNA (fuchsia, medium slate blue, and pale goldenrod), and DNA polymerase (medium slate blue, slate gray, light salmon, pale goldenrod) domains (D) Ribbon structure of the dimer of PF-8 with RTA (black) binding domain highlighted. (E) The dimer of PF-8 (cornflower blue) aligned with the dimer of BMRF1 (dim gray). (F) The surface and ribbon structure of the tetramer of BMRF1 aligned with the dimer ribbon structure of PF-8. All structures of PF-8 lack C-terminal residues 306–396. Structures were modeled with ChimeraX. dsDNA, double stranded DNA; PF, processivity factor; RTA, replication and transcription activator.

PF-8 consists of 396 amino acids and the crystal structure was solved at 2.8 Å resolution through an *Escherichia coli* expressed PF-8 in 2009. The crystal structure lacks residues 305–396 because it was predicted that residues 302–396 were unstructured, and the region was not necessary for DNA or DNA polymerase binding or processivity function in vitro. The crystal structure revealed that monomers exhibit a fold that is common among PFs and can form head-to-head homodimers.^41^ In addition to the crystal structure, the different domains or regions within PF-8 were determined experimentally with truncated mutants (Figure 2A–C). As shown in Figure 2A, the currently identified domains or regions include a nuclear localization sequence (NLS, 369–377) and two DNA polymerase interaction domains (10–27 and 277–304), dsDNA interaction domains (1–21 and 279–301), and dimerization domains (1–21 and 277–304).^34^ Several of the domains at least partially overlap with one another. The known PF-8 domains that play a critical role in lytic DNA replication are compelling targets to seek to disrupt and inhibit. Especially, if targeting one region will impact more than one of PF-8’s functions. Each domain is discussed in detail in the below sections.

### 3.2 | PF-8 dimers and nuclear import

The dimerization domains were identified through complementary in vitro and cellular experiments with full-length and truncated mutants. Chen et al. expressed full-length and truncated mutants with an IVT system. Compared to the full-length protein, two mutants with deletions at Δ277–396 and Δ1–21, respectively, did not produce bands consistent with a dimer while all other mutants generated and the full-length PF formed dimers, which suggests residues 1–21 and 277–304 were important for dimerization.^8^ Several years later, the crystal structure revealed that PF-8 dimerizes by stabilizing the interactions of residues in a loop and by hydrogen bonds between two strands that form an extended beta sheet. None of the residues involved in the formation of the loop, strands, or beta sheet are part of the dimerization domains previously identified, suggesting that deletion of residues 1–21 or 277–304 likely disrupts the overall protein conformation.^41^ While Zhou et al. transfected HeLa cells with several truncated PF-8 fusion mutants and observed similar results to Chen et al., to which truncated mutants formed dimers. Additionally, the full-length protein localized to the nucleus and Δ366–396 localized to the cytoplasm, but when the full-length and Δ366–396 were cotransfected the complex dimerized and mainly resided in the nucleus. Furthermore, mutants Δ366–396 and Δ1–21 were cotransfected and Δ366–396 localized to the cytoplasm while Δ1–21 localized to the nucleus suggesting that dimers likely form in the cytoplasm before translocating into the nucleus.^42^ Since lytic DNA replication occurs in the nucleus, PF-8 needs to translocate into the nucleus to bind to the oriLyt and/or associate with other viral DNA replication proteins for efficient DNA replication to occur. The dimerization domains may be essential for PF-8 to localize to the nucleus. Furthermore, the dimerization of PF-8 could be one of the first steps involved in forming the preinitiation complex.^42^ A potential antiviral target could be aimed at disrupting the formation of dimers in the cytoplasm and thus preventing PF-8’s processivity function in the nucleus.

### 3.3 | Oligomeric state of PF-8

Some of the PFs of human herpesviruses are known to exist in a higher oligomer state such as cytomegalovirus, EBV, and KSHV.^23^ Several studies have investigated the oligomer state of PF-8 with proteins generated from a variety of expression systems and approaches. Chen at el. expressed PF-8 in an *E. coli* system and through a gel filtration experiment revealed that it exists mostly as a dimer. Furthermore, PF-8 was expressed in an *E. coli* and an IVT system to produce bands that corresponded to protein monomers and monomers and dimers when crosslinked. In a Blue Native PAGE gel with PF-8 expressed from *E. coli* revealed that PF-8 could form monomers and dimers in the presence or absence of crosslinking.^8^

Although bacterial and cell free systems provide valuable information about proteins, mammalian expression systems may provide a more translatable system to study KSHV proteins. Gutierrez et al. transfected PF-8 into 293 L cells to assess oligomeric state. All lysates irrespective of conditions revealed PF-8 monomers. PF-8 from crosslinked lysate in the presence or absence EtBr or DNase formed tetramers. Furthermore, PF-8 and several truncated mutants expressed in iSLK BAC16 cells were detected as monomers, while the PF-8 that was crosslinked formed tetramers. Additionally, only monomers were observed when PF-8 samples were treated with DNase. Perhaps, PF-8 is stable enough to form tetramers when bound to DNA, but once DNase digestion shortens the length of the DNA fragment, PF-8 oligomers are no longer stabilized and dissociate in the absence of crosslinkers. Several mutants (Δ2–50, Δ51–100, Δ101–150, Δ151–200, Δ201–250, and Δ251–300) were shown to form monomers; while mutants Δ301–350 and Δ351–396 formed monomers and dimers when crosslinked.^32^ In addition, PF-8 from the nuclear extracts from reactivated BCBL-1 cells formed monomers and dimers when crosslinked.^8^

In summary, the differing conclusions regarding the oligomeric state of PF-8 could be due to the different experimental systems and methods. A common finding across all systems were that crosslinking preserved higher molecular weight oligomers. One limitation of crosslinking experiments using cell lysates versus purified proteins is the uncertainty of the protein(s) that are being crosslinked. Many crosslinking reagents work by covalently attaching two functional groups together. Crosslinking reagents can vary in spacer arm length ranging from zero to >100 Å. Longer spacer arms are typically more flexible and provide less steric hinderance but a drawback to using them is it increases the number of sites for nonspecific interactions to occur.^43^ It is reasonable to assume that the greater the spacer length the greater the possibility that the protein of interest is crosslinked through a nonspecific interaction^44^ Alternatively, there could be some level of equilibrium among the oligomeric states, and these may be stabilized by the presence of additional viral or cellular components or environmental conditions (e.g., molecular crowding, DNA, etc.). In addition, it is possible that the different oligomeric states of PF-8 carry out different functions. For example, the PF-8 dimers are responsible for processivity function while the tetramer plays more of a role in interacting with cellular proteins to evade immune responses. Further studies are needed to decipher the functionality of monomers and oligomers within infected cells.

## 4 | PF-8 LYTIC ORIGIN DNA BINDING SITES

PF-8 is known to interact with DNA and has two dsDNA binding domains between residues 1–21 and 279–301. PF-8 expressed via an IVT system and incubated with a ssDNA or dsDNA cellulose columns showed that PF-8 binds to dsDNA with five times greater affinity than ssDNA.^45^ Since PF-8 has a higher affinity for dsDNA that could indicate that PF-8 is one of the early DNA replication proteins to bind to the oriLyt. An electro-phoretic mobility shift assay (EMSA) with purified PF-8 from *E. coli* demonstrated that PF-8 binds DNA.^8^ Furthermore, several truncated mutants were expressed with an IVT system and applied to a dsDNA cellulose column. Mutants Δ359–396, Δ323–396, and Δ302–396 eluted at the same salt concentration as the full-length indicating similar affinity for dsDNA. The Δ279–396 mutant eluted at a lower concentration than the full-length indicating a lower affinity. Mutants Δ1–9, Δ1–27, and Δ1–62 eluted at varying concentrations.^45^ Chen et al. conducted a similar experiment but obtained slightly different results. Truncated mutants Δ277–396 and Δ1–21 did not bind to dsDNA. Mutants Δ371–396 and Δ305–396 eluted at the same salt concentration as the full-length protein which corresponds with previous results,^8^ confirming residues 1–21 and 279–−301 are likely important for PF-8 binding to dsDNA.^45^ In addition, an EMSA with nuclear extract from latent and reactivated BCBL-1 cells verified that PF-8 binds to DNA. There was a band shift, in the reactivated cells, that migrated approximately as far as the *E. coli* purified PF-8 with DNA. To further validate that PF-8 DNA binding activity, the EMSA was transferred to a membrane and probed with anti-PF-8, revealing only one band present in the reactivated sample with DNA. A limitation of the assay was that PF-8 unbound to DNA was not detected, likely due to of its high isoelectric point preventing it from entering the gel.^8^ To identify the DNA sequence(s) that PF-8 binds, ChIP assays using HEK293 cells infected with KSHV BAC36 found that PF-8 bound to the C/EBPα and RRE regions within the oriLyt.^34^ Future studies are needed to determine if there are other sites within the oriLyts and to correlate PF-8 oligomeric state with specific DNA-binding locations. Inhibiting PF-8’s interaction with DNA could be a worthwhile approach for antiviral development since it will lead to inefficient replication of the genome by preventing PF-8 binding and replisome assembly at the pre-initiation site. Other PFs are known to have transcriptional activity, but limited research has investigated PF-8’s role in this capacity. If it is discovered that PF-8 has transcriptional activity it introduces another possible mechanism for a multi-hit antiviral target that may diminish the formation of new virions by disrupting genome replication and altering gene expression.

## 5 | PF-8 BINDING DNA POLYMERASE PROMOTES NUCLEAR LOCALIZATION AND POLYMERASE PROCESSIVITY

The KSHV DNA polymerase’s primary role in lytic DNA replication is to incorporate nucleotides to synthesize new viral genomes copies. DNA polymerase is considered an early gene, is about 114 kDa, and consists of 1012 amino acids.^22,29^ Since lytic DNA replication occurs in the nucleus, DNA polymerase must first be transported from the cytoplasm to inside the nucleus. The KSHV DNA polymerase does not encode an NLS, so it requires another mechanism to enter the nucleus. To investigate whether PF-8 is responsible for transporting DNA polymerase into the nucleus, PF-8 was fused at the C-terminal with GFP and DNA polymerase with a Flag tag at the N-terminal and subsequently transfected into Vero cells.^46^ Utilizing confocal immunofluorescence microscopy approach, PF-8 was shown to localize to the nucleus and DNA polymerase to the cytoplasm. When PF-8 and DNA polymerase were cotransfected, they localized to the nucleus but when a truncated mutant lacking the NLS was cotransfected with DNA polymerase they localized to the cytoplasm. Furthermore, when either mutant Δ1–270 or Δ22–365 was cotransfected with DNA polymerase they localized to the nucleus. Residues 366–396 are crucial for nuclear localization of PF-8 and DNA polymerase.^46^ Chen et al. showed that PF-8 and DNA polymerase colocalizes in 293 T cells.^47^ These results are evidence that PF-8 and DNA polymerase associate in cytoplasm and are transported into the nucleus to form subsequence complexes with the other DNA replication proteins.

To identify the protein regions of PF-8 that are necessary for interaction with DNA polymerase a series of truncated mutants were examined. PF-8 was expressed as a fusion in a bacterial and IVT system to initially demonstration that PF-8 interacts with DNA polymerase.^22,45^ Further characterization of the residues necessary for DNA polymerase binding were tested by a co-IP assay. Full-length and mutants Δ302–396, Δ323–396, Δ359–396, and Δ1–9 were able to bind with DNA polymerase, while Δ1–27, Δ1–62, and Δ1–127 could not. Initial experiments could not rule out the importance of the C-terminal domains, due to the lack of an antibody that could recognize mutants Δ191–396, Δ234–396, and Δ279–396.^45^ However, related experiments were conducted with an antibody that could detect all the truncated mutants. Full-length, Δ371–396, and Δ305–396 were able to coprecipitate with DNA polymerase while Δ277–396 and Δ1–21 did not associate.^8^ Together these results suggest that residues 10–27 and 277–304 are important for DNA polymerase binding.

The primary function of PF-8 is to help keep DNA polymerase bound to DNA so efficient replication can occur. To assess the processivity function of PF-8, DNA polymerase was incubated with and without IVT expressed PF-8 and assayed using an in vitro DNA replication assay. When PF-8 was present, thousands of nucleotides could be incorporated, but only three nucleotides were incorporated when PF-8 was absent, indicating the inefficiency of KSHV DNA polymerase alone.^22^ PF-8 mutants Δ277–396 and Δ1–21 did not promote efficient processivity function while Δ371–396 and Δ305–396 retained processivity function.^8^ Additional studies of PF-8 truncation mutants, including Δ302–396, Δ323–396, and Δ359–396, which were shown to retain processivity function; while Δ1–27, Δ1–62, Δ1–127, Δ191–396, Δ234–396, and Δ279–396 lost processivity function. The Δ1–9 mutant had a less efficient processivity function compared to the full-length. Thus, residues 10–21 and 279–301 are likely important for processivity function.^45^ Furthermore, the full-length and mutant PFs’ ability to promote the replication of the KSHV genome in iSLK BAC16 cells were examined using qPCR. The full-length PF-8 efficiently replicated the viral genome and Δ301–350 and Δ351–396 at a decreased level. Mutants Δ2–50, Δ51–100, Δ101–150, Δ151–200, Δ201–250, and Δ251–300 were unable to promote synthesis of viral DNA.^32^ These results indicate that both of PF-8’s DNA polymerase domains are needed to promote efficient viral DNA synthesis and without PF-8’s association with DNA polymerase the viral genome will not be synthesized efficiently which will negatively affect the assembly of virions.

Interestingly, the deletion of one of PF-8’s DNA polymerase binding domains permitted transport of KSHV DNA polymerase into the nucleus, so perhaps the two DNA polymerase domains serve different functions. Another area that still needs to be explored is the monomeric or oligomeric state(s) that are necessary for PF-8 to interact with DNA polymerase. A promising antiviral target could be one that disrupts the interaction between PF-8 and DNA polymerase, which will ultimately effect DNA replication by preventing DNA polymerase from localizing to the nucleus. Without DNA polymerase in the nucleus, lytic DNA replication will not occur, and no new infectious virions will be produced.

## 6 | PF-8’S ASSOCIATION WITH LYTIC PROTEIN, RTA

RTA, an essential lytic protein, is responsible for the switch from latent to lytic and activates the expression of delayed early genes.^48^ It has been suggested that RTA is involved in forming the pre-initiation complex by recruiting viral proteins to the site of replication, such as PF-8. A co-IP assay with HEK293 cells cotransfected with PF-8 and RTA determined that RTA binds to PF-8. In addition, a co-IP assay with the lysate from BCBL-1 cells further validated RTA-PF-8 interactions. To elucidate the region that RTA interacts with PF-8, truncated mutants were cotransfected in HEK293 cells with RTA. Mutants Δ1–198 and Δ1–265 were able to bind with RTA while Δ199–396 could not. The region that is likely important for RTA binding to PF-8 is between residues 266–396. In addition, a mutant of PF-8 that lacked both dimerization domains was cotransfected with RTA to conclude that dimerization of PF-8 is not necessary for binding to RTA.^34^ To establish if RTA was necessary for PF-8 to bind to the KHSV oriLyt, anti-flag PF-8 was cotransfected with the oriLyt in the presence or absence of RTA in Vero cells. A ChIP assay was subsequently done with anti-flag and primers amplifying the C/EBP region of the oriLyt. When RTA was present, there was a more intense band compared to without it suggesting that RTA helps promote PF-8 binding to the C/EBPα region. A replication assay which included all the DNA replication proteins determined if both RTA and PF-8 were a requisite for lytic DNA synthesis. When all proteins were included, there was successful DNA synthesis, but when PF-8 was absent there was no DNA replication. In addition, when the replication assay included a mutant of RTA (350–550: interaction domain of PF-8) DNA replication was decreased.^34^ These results demonstrate that the protein-protein interaction between RTA and PF-8 are necessary for efficient lytic DNA replication. Disrupting the interactions between PF-8 and RTA may be a prospective approach for antiviral development since it would disrupt the pre-initiation complex from forming.

## 7 | GAMMA-HERPESVIRUS PFS: KSHV’S PF-8 AND EBV’S BMRF1

There are minimal studies for the PFs for MHV-68, HVS, and RRV. Much research is focused on EBV’s PF (BMRF1). EBV is known to cause mononucleosis, Burkitt’s lymphoma, Hodgkin lymphoma, and post-transplant lymphoproliferative.^49^ BMRFI shows several similarities to PF-8. Likewise, to the crystal structure of PF-8, BMRF1 lacks a portion of the C-terminal region because it is unstructured.^50^ Similarly, the C-terminal region does not affect processivity function or binding to DNA polymerase. BMRF1 has also been shown to exhibit multiple oligomeric states. The crystal structure for BMRF1 revealed that it can form head-head homodimers (Figure 2E) but can also form a tetramer ring structure through tail-to-tail association (Figure 2F).^50–52^ As shown in Figure 2E, some regions of PF-8 and BMRF1 align perfectly (some beta sheets and helices) while some regions are slightly off centered (loops), but overall, they share numerous secondary structures. Furthermore, negative staining with transmission election microscopy (TEM) showed that BMRF1 exhibited a ring structure and is hypothesized to be a hexamer.^51^ Since BMRF1 exhibits higher multimeric structures, PF-8 may also form them. Likewise, to PF-8 BMRF1 binds to dsDNA at a higher affinity compared to ssDNA and has two dsDNA binding domains.^53–55^ BMRF1 also interacts with DNA polymerase and when present increases the rate of polymerization.^53^ BMRF1 similarly to PF-8 localizes to the nucleus and helps transport DNA polymerase there.^56,57^ Also, BMRF1 is crucial for lytic DNA replication since deletion of it in the genome resulted in the virus unable to be replicated.^58^ Table 1 shows that BMRF1 shares low sequence similarity with PF-8 when comparing the entire protein sequence or specific domains. While Figure 1 reveals highly conserved amino acids visible in amino acid alignment, especially across residues color coded by residue chemical property. Additionally, the similar structural folds and functions suggest conservation at the protein function level (Figure 2E). By studying the similarities and difference of BMRF1 and PF-8, it could lead to a more comprehensive picture of PFs, and increase the likelihood of discovery of pan-antiviral targets.

## 8 | PF-8 AS A VIABLE ANTI-VIRAL TARGET

Most herpesvirus antiviral drugs that are clinically available focus on the DNA replication process through either targeting DNA polymerase/thymidine kinase or the helicase-primase.^59,60^ One of the biggest hurdles with discovering effective antivirals for KSHV is that the results from in vitro systems do not mimic the clinical trials results. For example, DNA polymerase targets such as ganciclovir and foscarnet have been effective in inhibiting KSHV DNA replication in cell culture but were ineffective in treating patients with KS lesions.^7^ According to clinicaltrials.gov there have been over 50 drugs that have been used in clinical trials to try and develop treatments against KSHV and KSHV-associated diseases. Various drug classes have been investigated in clinical trials such as chemotherapeutics, kinase inhibitors, protease inhibitors, and antibodies. In addition, 12 of candidates are within the subclasses of nucleoside reverse transcriptase inhibitors, non-nucleoside reverse transcriptase inhibitors, and nucleoside analogs all of which target DNA replication. With the unsatisfactory performance and/or efficacy of the current antivirals available new antivirals are needed.^61^

**FIGURE 3:**
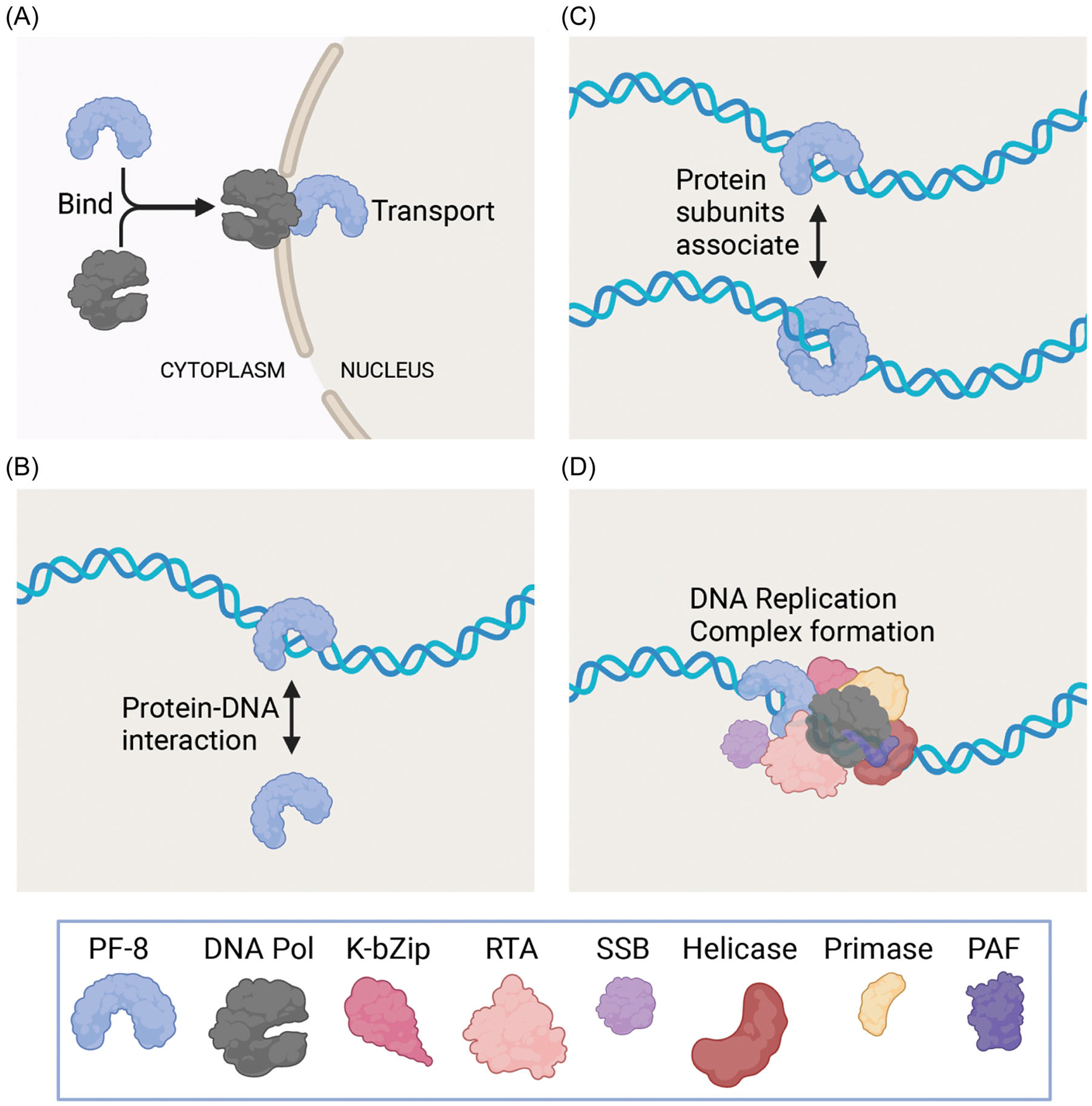
Molecular interactions that govern PF-8 to functions. By inhibiting one or several of the known protein-protein and protein-DNA binding of PF-8, KSHV DNA replication and subsequently the generation of new virions may be effectively blocked. (A) PF-8 binds and translocated DNA polymerase into the nucleus. (B) PF-8 binds dsDNA. (C) PF-8 complexes bind dsDNA. (D) The KSHV DNA replication complex forms on dsDNA to initiate DNA replication. Created with Biorender. dsDNA, kilobase double stranded DNA. dsDNA, double stranded DNA; KSHV, Kaposi’s Sarcoma Herpesvirus; PF, processivity factor.

One approach to discovering new antivirals is to investigate other mechanisms to inhibit lytic DNA replication such as by pursuing inhibitors that limit the function of PF-8. For example, Dorjsuren et al. discovered a pyrimidoquinoline analog that was able to inhibit DNA synthesis in vitro and reduce lytic DNA replication in cells by targeting the DNA polymerase/PF-8 complex.^21^ PF-8 represents an optimal antiviral target since it has multiple functions and binding partners during lytic DNA replication. Inhibition of one or multiple of PF-8 functions would diminish viral replication and new virion production. Due to overlapping domains (Figure 2A) there may be one inhibitor that may prevent more than one of the PF-8 activities, thereby more effectively blocking assembly of new infectious virus particles. Figure 3 summarizes PF-8’s known protein–protein and protein-DNA interactions that are essential for successful viral replication. Disrupting the DNA polymerase and PF-8 binding would prevent translocation of KSHV DNA polymerase into the nucleus and prevent new copies of the KSHV genome from being synthesized (Figure 3A). Interfering with DNA and homo-oligomerization of PF-8 would decrease DNA-binding activities that would prevent DNA replication and possible transactivation functions of PF-8 (Figure 3B,C). Blocking the formation of the lytic DNA replication complex by disrupting PF-8 binding to RTA (Figure 3D) would inhibit viral DNA replication and subsequent assembly and egress of new infectious virions.

## 9 | CONCLUSIONS AND PERSPECTIVES

PF-8 has several essential activities and interacts with various KSHV proteins and viral DNA to promote efficient viral replication and new virion production. PF-8 deletion mutants were critical to map the protein domains (Figure 2A). However, future studies using a more directed approach to mutate specific protein residues will elucidate key amino acids responsible for PF-8’s functional domains and also increase the probability of fully folded PF-8. Further research to examine the DNA and protein–protein interactions directly and assess the heterogeneity within the population of PF-8 will add missing details to elucidate PF-8’s activities. To accomplish this objective, microscopy approaches and direct visualization would continue to build the picture of PF-8’s protein–protein and protein-nucleic acid complexes. More specifically, cryo-EM would permit high resolution structures of full-length PF-8 in the presence and absence of DNA to provide additionally clarity of the role of the C-termini region in the formation of protein dimers, tetramers or higher molecular weight oligomers.^62–64^ Single molecular TEM studies comparing wild-type and PF-8 mutants in the presence of viral DNA sequences will delineate the minimal protein domains necessary for DNA-binding functions and elucidate the oligomeric state of PF-8 when bound to DNA locations.^36,65^ Studies using purified, full-length PF-8 in concert with other KSHV replication proteins may reveal that function is dependent on PF-8’s ability to bind with other KSHV proteins as well as form larger complexes. We have applied a TEM approach to examining the in vitro binding activity of purified KSHV RTA.^66^ We intend to complete an in-depth molecular study of purified PF-8 to evaluate whether the different oligomer states (monomer, dimer, tetramers, etc.) have unique roles or discrete DNA binding locations that would indicate DNA replication or potential transcriptional activity. The structural and molecular findings would inform downstream experiments in cell culture systems to assess the overall impact of preventing PF-8 functions on new virus production. With continued focus, more will be learned about the molecular interactions that control the viral replication process and increase the likelihood that effective antivirals will be developed against KSHV.

## Figures and Tables

**TABLE 1:** Gamma-herpesviruses processivity factor protein sequence percent similarities.

	Full-length				Residues 1–27				Residues 277–304			
	RRV	HVS	EBV	MHV-68	RRV	HVS	EBV	MHV-68	RRV	HVS	EBV	MHV-68
KSHV	52.2	30.0	25.1	25.5	66.7	29.0	12.9	16.7	45.2	29.4	20.0	37.5
RRV	-	29.8	25.8	24.4	-	29.0	25.8	20.0	-	32.4	17.1	34.4
HVS	-	-	27.4	23.8	-	-	32.3	22.6	-	-	8.6	22.9
EBV	-	-	-	23.1	-	-	-	30.8	-	-	-	14.3

Abbreviations: EBV, Epstein-Barr virus; HVS, Herpesvirus Saimiri; KSHV, Kaposi’s Sarcoma Herpesvirus; MHV-68, Murine Herpesvirus-68; RRV, Rhesus Macaque Rhadinovirus.

## Data Availability

The data that support the findings of this study are available in GenBank at https://www.ncbi.nlm.nih.gov/genbank/, reference number GQ994935.1, AAD21393.1, CAA45682.1, CAA24844.1, AAB66449.1. These data were derived from the following resources available in the public domain:—GenBank, https://www.ncbi.nlm.nih.gov/nuccore/GQ994935.1/—GenPept, https://www.ncbi.nlm.nih.gov/protein/AAD21393.1—GenPept, https://www.ncbi.nlm.nih.gov/protein/CAA45682.1—GenPept, https://www.ncbi.nlm.nih.gov/protein/CAA24844.1—GenPept, https://www.ncbi.nlm.nih.gov/protein/AAB66449.1.
